# Dietary Spirulina effects in *Eimeria*-challenged broiler chickens: growth performance, nutrient digestibility, intestinal morphology, serum biomarkers, and gene expression

**DOI:** 10.1093/jas/skae186

**Published:** 2024-07-12

**Authors:** Emmanuel Oluwabukunmi Alagbe, Hagen Schulze, Olayiwola Adeola

**Affiliations:** Department of Animal Sciences, Purdue University, West Lafayette, IN, USA; Livalta, an AB Agri Company, Peterborough, United Kingdom; Department of Animal Sciences, Purdue University, West Lafayette, IN, USA

**Keywords:** antioxidant, coccidiosis, feed additive, intestinal histomorphology, spirulina

## Abstract

This study investigated the growth performance, nutrient utilization, and intestinal health responses of *Eimeria*-challenged broiler chickens to dietary Spirulina (*Arthrospira platensis*). On day 1, birds were assigned to 2 diets supplemented with Spirulina (0 or 5 g/kg) in a randomized complete block design. The birds within each diet were divided into 2 *Eimeria*-challenge groups (challenge or no-challenge) and that resulted in a 2 × 2 factorial arrangement with 2 levels each of Spirulina and challenge on day 14. On day 15, the birds in the challenge or no-challenge groups were orally gavaged with a solution containing *Eimeria* oocysts or 1% PBS, respectively. Samples were collected on days 21 and 26 (6- and 11-d post-infection; **dpi**). Data collected from days 1 to 26 were analyzed using the MIXED procedure of SAS. Birds that were fed Spirulina-supplemented diets had increased (*P* < 0.05) BW gain, gain-to-feed ratio, and total tract retention nitrogen from days 14 to 21. The ileal villus perimeter and area, serum catalase, HMOX1 and SOD1 jejunal abundance were all increased (*P* < 0.05) in birds fed Spirulina-supplemented diets on day 21 (6 dpi). However, there was no effect on bone ash or oocyst count. From days 21 to 26, there was a tendency (*P* = 0.059) for a Spirulina × Challenge interaction on the BW gain of birds. Moreover, dietary Spirulina addition increased (*P* < 0.05) serum catalase, total antioxidant capacity, ileal villus perimeter, tibia bone ash, and the relative mRNA expression of HMOX1, SOD1, claudin 1, and TNFα in the jejunal mucosa of birds on day 26 (11 dpi). On both 6 and 11 dpi, the *Eimeria* challenge negatively (*P* < 0.05) impacted growth performance, gut morphology, and the relative mRNA expression of genes. Overall, assessing the impact of Spirulina in broilers revealed its positive antioxidant, immune-modulating, and health benefits. However, its dietary addition did not completely reverse the *Eimeria*-induced effects in these birds. Ultimately, this study outlines the positive properties of dietary Spirulina beyond its use in the diet of healthy broiler chickens.

## Teaser

Investigating the effect of dietary Spirulina supplementation on *Eimeria*-challenged birds demonstrated its favorable antioxidant, immune-modulating, and health benefits. Nevertheless, more studies are needed to understand its mode of action in reducing oocyst shedding and regulating gut health in broiler chickens.

## Introduction

Poultry meat products provide affordable and high-quality animal protein for human consumption, with broiler chickens being at the forefront of this industry (Petracci et al., 2013). Nevertheless, the poultry industry faces several threats that may undermine poultry health and thus meat production, leading to economic loss. One of the most prominent challenges to the poultry industry is coccidiosis, which accounts for approximately $13 billion in global losses (Dalloul and Lillehoj, 2006; Blake et al., 2020). Coccidiosis is a parasitic disease caused by the apicomplexan protozoans of the genus *Eimeria*. It leads to impaired nutrient absorption, frangible bones, reduced growth efficiency, inflammation, and intestinal damage in birds (Osho et al., 2019; Alagbe et al., 2023; Tompkins et al., 2023).

For many years, the conventional approach to managing coccidiosis in broiler chickens has heavily relied on anticoccidial drugs and prophylactic coccidiostats. While these drugs have been largely effective in controlling coccidiosis, the poultry industry has been under constant pressure to reduce the use of these drugs (Allen and Fetterer, 2002; Dalloul and Lillehoj, 2006). This is mostly due to concerns about the spread of resistant *Eimeria* spp. and public health unease about drug residue in poultry products (Allen and Fetterer, 2002). In recent years, there has been an emphasis on nutritional strategies to mitigate the negative effect of coccidiosis in broiler chickens, including the use of feed additives, amino acid supplementation, and mineral addition (Kiarie et al., 2019; Liu et al., 2023; Teng et al., 2023).

Spirulina (*Arthrospira platensis*) is a single-celled cyanobacterium that is rich in bioactive compounds, including phycocyanin, polysaccharides, B vitamins, minerals, polyunsaturated fatty acids, and carotenoids. These bioactive compounds confer antioxidant, anti-inflammatory, and immunomodulatory properties to Spirulina (Wu et al., 2016; Fries-Craft et al., 2021; Han et al., 2021). While its use as a health supplement in humans has been widely explored, its application in animals is just receiving nascent attention (Mazo et al., 2004). Previous studies have demonstrated the potential of Spirulina to alleviate the production of stress hormones, restore performance, and enhance antioxidative activities in heat-stressed birds (Mirzaie et al., 2018; Moustafa et al., 2021; Mullenix et al., 2021). Its positive effects on modulating the immune response and intestinal integrity of birds have also been reported. Moreover, Spirulina might have beneficial effects on the antioxidative system; it may also help resolve systemic inflammation in birds fed low-protein diets (Mullenix et al., 2021; Abdel-Moneim et al., 2022).

Despite the potential benefits of Spirulina, there remains a substantial gap regarding the effects of Spirulina on the performance, nutrient digestibility, bone mineral deposition, and intestinal health of *Eimeria*-challenged broiler chickens. We hypothesized that an *Eimeria* challenge would reduce the performance, nutrient digestibility, bone mineral deposition, and intestinal integrity of birds and that dietary Spirulina supplementation, through its immunomodulatory, antioxidant, and anti-inflammatory properties, would reduce the *Eimeria*-associated disruption in intestinal integrity and structure and thus ameliorate the *Eimeria*-induced effects in birds. Therefore, this study was designed to investigate the effect of Spirulina on the performance, nutrient digestibility, immune response, bone mineral deposition, and serum biomarkers in *Eimeria*-challenged broiler chickens.

## Materials and Methods

### Animals, diets, experimental design, and *Eimeria* challenge

The Purdue University Animal Care and Use Committee (West Lafayette, IN) approved the protocol used in the study.

A total of 384 male broiler chickens (Cobb 500), obtained from a commercial hatchery (Cobb-Vantress Inc., Siloam Springs, AR, USA), were used for this experiment. On day 0, the birds were housed in electrically heated 0.35 m^2^ battery brooders (model SB 4 T; Alternative Design Manufacturing, Siloam Springs, AR, USA) and fed a corn–soybean meal-based starter diet. The birds were individually weighed and tagged with identification numbers using a multi-PIT tag injector (UI Devices, Lake Villa, IL, USA). On day 1, the diets were changed to experimental diets. Birds had ad libitum access to feed and water during the experimental period. The experimental diets were provided in mash form and consisted of a corn–soybean meal-based diet supplemented with Spirulina (Organic Spirulina; RFI Ingredients, Broomfield, CO, USA) at 0 or 5 g/kg (Table 1). All experimental diets were formulated to meet or exceed the recommendations outlined in the Cobb 500 management guide for the grower phase (Cobb-Vantress, 2016). Research indicates that in poultry, alterations to feed formulation could potentially induce inflammation in the gastrointestinal tract (Cardoso Dal Pont et al., 2020). Hence, to mitigate any potential impact of the basal diet on intestinal health status, a consistent nutritional level was maintained for the experimental duration. Nevertheless, it is important to note that this was one of the limitations of this experiment. Diets were free from antibiotics or coccidiostats, and titanium dioxide was added to all diets at 5 g/kg as an indigestible marker.

**Table 1.** Ingredient and nutrient composition of diets, as-fed basis

Item	Spirulina, g/kg	
	0	5
Ingredient, g/kg		
Corn	589.5	539.5
Soybean meal (48% CP)	302.0	302.0
Soybean oil	45.0	45.0
Ground limestone	19.0	19.0
Monocalcium phosphate	11.0	11.0
Salt	4.0	4.0
dl-Methionine	1.2	1.2
l-Lysine HCL	0.3	0.3
l-Threonine	0.1	0.1
Vitamin–mineral premix^1^	3.0	3.0
Spirulina premix^2^	–	50.0
Titanium dioxide premix^3^	25.0	25.0
Total	1,000.0	1,000.0
Calculated nutrient and energy		
ME, kcal/kg	3,108	3,108
CP, %	20	20
Ca, g/kg	9.8	9.8
P, g/kg	6.1	6.1
Non-phytate P, g/kg	4.0	4.0
Amino acids		
Arg	12.7	12.7
Met	4.3	4.3
Ile	8.2	8.2
Lys	10.7	10.7
Thr	7.4	7.4
Trp	2.4	2.4
Met + Cys	8.8	8.8
Val	9.1	9.1
Analyzed nutrient and energy		
Gross energy, kcal/kg	3,900	3,960
CP, %	19	19
P, g/kg	6.1	6.1

^1^Provided the following quantities per kilogram of complete diet: vitamin A, 5,484 IU; vitamin D_3_, 2,643 ICU; vitamin E, 11 IU; menadione sodium bisulfite, 4.38 mg; riboflavin, 5.49 mg; d-pantothenic acid, 11 mg; niacin, 44.1 mg; choline chloride, 771 mg; vitamin B_12_, 13.2 μg; biotin, 55.2 μg; thiamin mononitrate, 2.2 mg; folic acid, 990 μg; pyridoxine hydrochloride, 3.3 mg; I, 1.11 mg; Mn, 66.06 mg; Cu, 4.44 mg; Fe, 44.1 mg; Zn, 44.1 mg; Se, 300 μg.

^2^5 g Spirulina powder added to 45 g of corn to make 50 g Spirulina premix. 50 g/kg of Spirulina premix delivered 5 g/kg of Spirulina. The composition of the Spirulina used in this study are as follows: 12 mg/g chlorophyl, 4.5 mg/g carotenoids, 160 mg/g crude phycocyanin, 650 g/kg crude protein.

^3^Prepared as 5 g TiO_2_ plus 20 g corn.

From day 1, the birds were assigned to the 2 diets supplemented with Spirulina at 0 or 5 g/kg in a randomized complete block design, and BW was used as a blocking factor. Each dietary treatment consisted of 16 replicate cages and 12 birds per cage for a total of 192 birds per diet. On day 14, chickens were individually weighed, and leftover feed was weighed to estimate feed intake (**FI**). Furthermore, all birds within each of the 0 or 5 g/kg Spirulina diets were pooled and re-randomized. This was done to equalize the average BW. With this re-randomization, the number of birds was reduced to 10 birds per cage, and each of the 2 diet groups was divided into 2 challenge groups (challenge or no-challenge), resulting in 4 experimental treatments. The study was set up as a 2 × 2 factorial arrangement of treatments with 2 experimental diets (0 or 5 g/kg Spirulina) and 2 *Eimeria*-challenge states (challenge or no-challenge) comprising 8 replicate cages and 10 birds per cage. Birds in both challenge groups were housed in the same environmentally controlled room. However, the challenge group was isolated from the no-challenge group to avoid cross-contamination.

**Figure 1.**
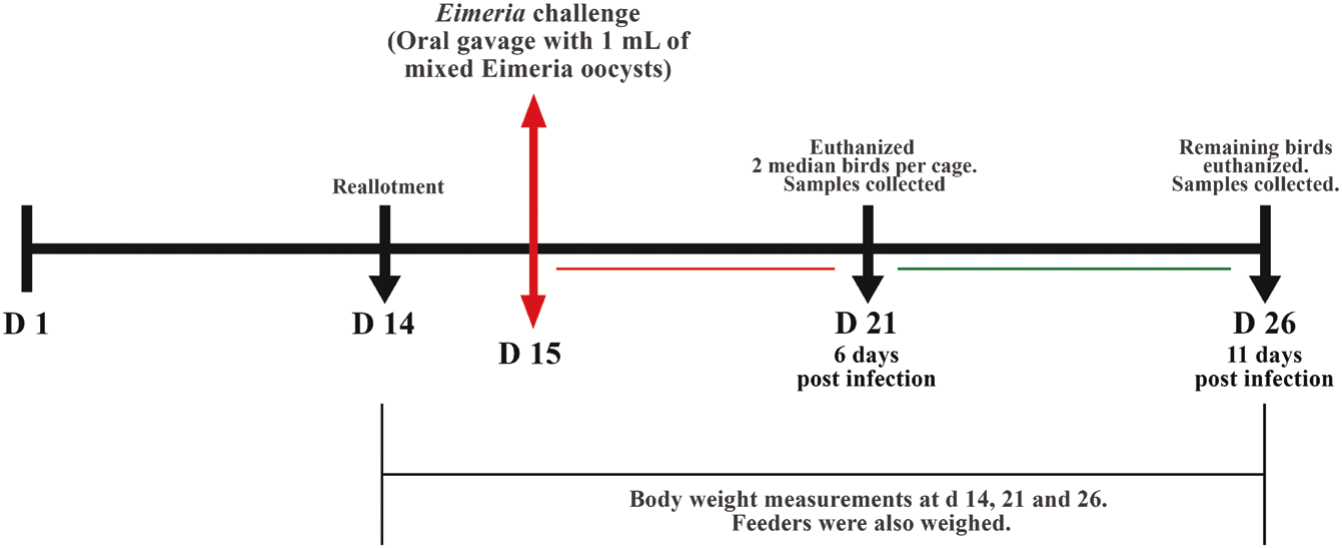
Diagram depicting the experimental procedure. Broiler chickens were fed experimental diets supplemented with 0 or 5 g/kg Spirulina from days 1 to 26. On day 14, the birds were reallotted and separated into a challenge and no-challenge group within each diet. On day 15, birds in the challenge or no-challenge groups were orally gavaged with 1 mL of *Eimeria* oocysts or 1% PBS, respectively. Growth performance was measured from days 14 to 26. Excreta, blood, and intestinal tissue were collected on day 21 (6 dpi). Excreta, blood, intestinal tissue, and ileal digesta samples were collected on day 26 (11 dpi).

On day 15, the birds in the challenge group were orally gavaged with 1 mL solution containing 25,000, 25,000, and 125,000 oocysts of *E. maxima*, *E. tenella*, and *E. acervulina*, respectively. This dosage was chosen according to our previous research (Alagbe et al., 2023). In the no-challenge group, the birds received 1 mL of 1% PBS (VWR International, Radnor, PA, USA) through an oral gavage. Careful consideration was given to handling feed and excreta to prevent the transfer of oocysts. The experiment lasted 25 d from days 1 to 26 (Figure 1).

### Growth performance, sample collection, and processing

The feeder weight and individual BW of birds were recorded on days 14, 21, and 26. The BW gain, FI, and gain-to-feed ratio (**G:F**) were determined from days 14 to 21 and from days 21 to 26. Mortality was recorded daily and when it occurred, it was used to correct G:F. Excreta samples on white paper-lined pans under each cage were collected from days 19 to 21, and pooled; and from days 24 to 26, and pooled. Excreta samples were stored at −20 °C until further analysis for eventual determination of the total tract retention (**TTR**) of nutrients. Fresh excreta samples were also collected from all cages into labeled 15 mL tubes (VWR International, Radnor, PA, USA) on day 21 (6 days post infection, **dpi**) and day 26 (11 dpi) for oocyst counting. Excreta samples for oocyst counting were mixed with a wooden spatula and stored at 4 °C.

On day 21 (6 dpi), the 2 median birds per cage were separated for sample collection. The first median bird was euthanized by CO_2_ asphyxiation, and intact jejunal and ileal tissues were collected (10 cm of the mid jejunum or mid ileum). The excised intestinal segments were rinsed with ice-cold 1% PBS, stapled to cutout cardboard, and placed in 10% buffered formalin (VWR International, Radnor, PA, USA) until processed. From the second median bird, blood (approximately 4 mL) was collected into non-heparinized tubes and centrifuged at 3,000 **× ***g* for 15 min at 4 °C after clotting. Serum was aspirated and stored at −80 °C until further analyses. Jejunal segments were excised from this same bird, flushed with cold PBS, cut longitudinally in half to expose the lumen, and mucosa was scraped with glass slides. Mucosal contents were placed in 1.5 mL of Trizol reagent (Invitrogen, Grand Island, NY, USA), rapidly frozen in liquid nitrogen, and transferred to a −80 °C freezer pending RT-PCR analysis. The left tibia from the 2 median birds was collected and stored at −20 °C pending bone mineral deposition analysis.

On day 26 (11 dpi), 2 median birds per cage were separated for sample collection. Intact jejunal and ileal tissues were collected from the first median bird using the same procedure as on day 21 (6 dpi). Blood samples and jejunal mucosa for RT-PCR were also collected using the same procedures as on day 21 (6 dpi). Additionally, the left tibia from the 2 median birds was collected and stored at −20 °C pending bone mineral deposition analysis. The remaining birds on day 26 (11 dpi) were euthanized and ileum was removed. The distal two-thirds of the ileum was flushed with distilled water into pre-labeled plastic containers for ileal digesta collection. The samples were stored at −20 °C for subsequent determination of the apparent ileal digestibility (**AID**) of nutrients.

### Intestinal histomorphology

Purdue Histology and Phenotyping Laboratory processed and stained the jejunal and ileal tissue sections with Alcian blue and a nuclear fast red counter stain. Villus height, crypt depth, villus perimeter, and villus area were measured using a microscope with an electronic camera (National Optical and Scientific Instruments, Inc., Schertz, TX, USA) and an ImageJ macro (ImageJ open-source software version 1.8) as described by Alagbe et al. (2022). Villus height and crypt depth were measured from 5 villi per bird, and only intact and unbroken villi were considered. The villus height was defined as the distance from the tip of the villus to the crypt mouth, whereas crypt depth was defined as the distance from the base of the villus to the muscularis mucosa. Villus perimeter was defined as the length of the boundary of the villus, and it was measured by tracing around the villus with the freehand line selection tool in the ImageJ software. The villus area referred to the size of the surface of the villus. It was measured by capturing the entire enclosed area within the villus by using the same freehand selection tool, and the ImageJ software calculated the area based on the outlined region. Villus height to crypt depth ratio (**VH/CD**) was also calculated. Goblet cells were counted from the same 5 villi per bird, and the average was calculated.

### Oocyst counting

Approximately 2 g of excreta sample containing sporulated oocysts were added to 28 mL of magnesium sulfate (MgSO_4_) solution, which served as the flotation solution. This MgSO_4_ solution was prepared by dissolving 125 g of MgSO_4_ salt in 500 mL of double-distilled water. The sample in the flotation solution was homogenized using a wooden spatula and allowed to sit for 5 min. The mixture was then sieved twice using a fine-mesh kitchen sieve. Disposable plastic droppers (Fisher Scientific, Waltham, MA, USA) were used to carefully fill the chambers of a McMaster slide (Jorgensen Laboratories, Loveland, CO, USA). The slide was set on a level surface for 5 min, allowing the oocysts to rise to the surface. Afterward, the oocysts were counted under a microscope with an electronic camera. The results obtained were multiplied by a factor of 50 and expressed as the number of oocysts per gram of excreta.

### Total RNA extraction and reverse transcription

Total RNA was extracted from the jejunal mucosa stored in the Trizol reagent following the manufacturer’s protocol. The RNA concentrations were determined using the NanoDrop 1000 (Thermo Fisher Scientific, Waltham, MA, USA), and we verified RNA integrity using 1% agarose gel electrophoresis. Subsequently, 2 mg of total RNA from each sample were reverse transcribed into cDNA using the M-MLV reverse transcription reagent (Promega, Madison, WI, USA). The resulting cDNA was then diluted to a 1:10 ratio with nuclease-free water (Ambion, Austin, TX, USA) and stored at −80 °C for subsequent analyses.

### Quantitative real-time PCR analysis

Real-time PCR of heme oxygenase 1 (**HMOX1**), interleukin 1β (**IL-1β**), interleukin 6 (**IL-6**), superoxide dismutase type 1 (**SOD1**), tumor necrosis factor alpha (**TNFα**), and claudin 1 genes was conducted using the Bio-Rad CFX thermocycler (Bio-Rad, Temecula, CA, USA) with the SYBR real-time PCR mix (Biotool, Houston, TX, USA) in a total reaction volume of 20 μL. The PCR reactions were incubated for 3 min at 95 °C, after which samples were subjected to 40 cycles of an amplification protocol as follows: 95 °C for 10 s, primer-specific annealing temperature for 30 s, and 95 °C for 10 s. A melt curve analysis was performed for each gene after the PCR run. The annealing temperatures and primer sequences used are listed in Table 2. Samples were analyzed in duplicates, and the acceptable coefficient of variation was set at ≤5%. Relative gene expression was calculated using the 2^−ΔΔCt^ method (Livak and Schmittgen, 2001) with normalization against the housekeeping gene, glyceraldehyde-3-phosphate dehydrogenase (**GAPDH**).

**Table 2.** Sequences of primers used for the real-time PCR analysis

Target gene	Primer sequence (5ʹ to 3ʹ)	Annealing temperature (°C)	GenBank accession number
HMOX1	F: CTGGAGAAGGGTTGGCTTTCT	60.0	NM_205344
	R: GAAGCTCTGCCTTTGGCTGTA		
IL-1β	F: GCATCAAGGGCTACAAGCTC	57.7	NM_204524
	R: CAGGCGGTAGAAGATGAAGC		
IL-6	F: CCCTCACGGTCTTCTCCATA	53.0	NM_204628.1
	R: CTCCTCGCCAATCTGAAGTC		
SOD1	F: ATTACCGGCTTGTCTGATGG	58.0	NM_205064.1
	R: CCTCCCTTTGCAGTCACATT		
TNFα	F: AGATGGGAAGGGAATGAACC	55.7	AY765397
	R: ACTGGGCGGTCATAGAACAG		
Claudin 1	F: CAGACTCTAGGTTTTGCCTT	52.0	NM_001013611.2
	R: AATCTTTCCAGTGGCGATAC		
GAPDH	F: ATGACCACTGTCCATGCCATCA	59.0	NM_204305.1
	R: AGGGATGACTTTCCCTACAGCCTT		

HMOX1 = heme oxygenase 1; IL-1β = interleukin 1 beta; IL-6 = interleukin 6; SOD1 = superoxide dismutase 1; TNFα = tumor necrosis factor alpha; GAPDH = glyceraldehyde-3-phosphate dehydrogenase.

### Serum biomarkers

The activities of serum alkaline phosphatase (**ALP**) (Lot No. 701710, Cayman Chemical, Ann Arbor, MI, USA), catalase (Lot No. 707002, Cayman Chemical, Ann Arbor, MI, USA), and total antioxidant capacity (**TAC**) (Lot No. 709001, Cayman Chemical, Ann Arbor, MI, USA) were measured as recommended by the manufacturer.

### Bone-breaking strength and ash analysis

The attached tissue on the left tibia bones was removed with a scalpel. The weight, length, and width of the bones were measured. Bone-breaking strength of the tibia bones was measured via a 3-point bend test using a texture analyzer (TA.XTPlus Connect, Texture Technologies Corp., Hamilton, MA, USA). The generated result shows the plateau curve of force needed to break the bone expressed as energy stored in the tibia (Sung et al., 2024). The broken bones were defatted in a Soxhlet extractor using petroleum ether. They were dried, weighed, and ashed in a muffle furnace for 16 h at 600 °C to determine the bone ash following the method described by Walk et al. (2024).

### Chemical analysis

The experimental diets and dry excreta samples were ground using a centrifugal grinder (ZM 200; Retsch GmbH, Haan, Germany). The dry ileal digesta samples were ground using a coffee grinder. The ground samples were then dried at 105 °C in a forced-air drying oven (Precision Scientific Co., Chicago, IL, USA; method 934.01; AOAC, 2006) until a constant weight was achieved, enabling the determination of the dry matter (**DM**). Gross energy (**GE**) in the samples was analyzed using an isoperibol bomb calorimeter (Parr 6200; Parr Instrument Co., Moline, IL, USA), and nitrogen (N) content was determined using the combustion method (TruMac® N; LECO Corp., St. Joseph, MI, USA; method 990.03; AOAC, 2000). The concentration of titanium was measured at an absorbance of 410 nm using a microplate reader, following the procedure outlined by Myers et al. (2004).

### Calculations

Using the index method, the TTR or AID (%) of nutrients was calculated using the outlined equation Adeola (2001):

TTR or AID, % = 100 – [(Ti_I_/Ti_O_) × (D_O_/D_I_) × 100]

where Ti_I_ and Ti_O_ are the concentrations of titanium (g/kg DM) in diets, and excreta/ileal digesta samples, respectively; D_I_ and D_O_ are the concentration of nutrients (g/kg DM) in diets and excreta or ileal digesta samples, respectively.

The AID of GE and the apparent metabolizable energy (**AME**) of the diet were computed as a product of the coefficient and GE concentrations (kcal/kg) in the diet. Using a factor of 8.22 kcal/g N (Hill and Anderson, 1958), the nitrogen-corrected AME (**AMEn**) was computed by correcting for zero N retention following the method outlined by Zhang and Adeola (2017):

AMEn, kcal/kg DM = [AME, kcal/kg DM – (8.22 × N_rt_)]

where N_rt_ = N retention in g/kg of DM intake. The N_rt_ was calculated as outlined as follows:


$$N_{rt},g/kgDM=NI[N_{O}\times \left(Ti_{I}/Ti_{O}\right)]$$

where N_I_ and N_O_ are the N concentrations (g/kg DM) in the diet and excreta, respectively.

### Statistical analysis

Data were analyzed as a randomized complete block design using the MIXED procedure of SAS. A 2 × 2 factorial arrangement of Spirulina level (0 or 5 g/kg) and *Eimeria*-challenge state (challenge or no-challenge) was used. The main effects of Spirulina level and *Eimeria*-challenge state, as well as the interaction between these 2 factors, were tested and considered as the fixed effects. The replicate blocks were considered as random effects. Where interaction was significant, the Tukey test was used for mean separation. The initial BW of birds on day 14 was the blocking factor. Oocyst count data were log-transformed and analyzed for the effect of Spirulina on oocyst count in birds. Outliers, defined as values outside of ± 1.5 × interquartile range, were identified and removed. Statistical significance and tendency were declared at *P <* 0.05 and 0.05 ≤* P <* 0.10, respectively. Cage was considered as the experimental unit.

## Results

### Growth performance

The growth performance of birds is presented in Table 3. Regardless of the *Eimeria* challenge, dietary Spirulina supplementation increased (*P* < 0.05) the BW gain and G:F of birds from day 14 to 21. From days 21 to 26, dietary Spirulina increased (*P* < 0.01) the BW and showed a tendency (*P* = 0.07) to increase G:F of broiler chickens irrespective of the challenge state. There was also a tendency for a Spirulina × Challenge interaction (*P* = 0.059) on BW gain of birds. From days 14 to 26, Spirulina increased (*P* < 0.05) the BW gain and G:F of broiler chickens regardless of the *Eimeria* challenge; however, there was no effect on FI. The *Eimeria* challenge reduced (*P* < 0.01) the BW gain, FI, and G:F during days 14 to 21 and days 14 to 26. The same *Eimeria*-challenge effect was observed in birds during days 21 to 26, although G:F was unaffected.

**Table 3.** Growth performance of *Eimeria*-challenged broiler chickens fed Spirulina-supplemented diets, days 14 to 26^1^

Item	Spirulina		*Eimeria* challenge		SEM^2^	*P* value		
	0 g/kg	5 g/kg	No-challenge	Challenge		Spirulina	Challenge	Spirulina × Challenge
BW, g								
Day 14	433	432	432	433	19.90	–	–	–
Day 21	788	806	848	746	23.73	0.023	0.001	0.923
Day 26	1,173	1,224	1,270	1,127	28.69	0.001	0.001	0.133
Days 14 to 21								
BW gain, g/bird	355	373	415	313	5.46	0.014	0.001	0.961
Feed intake, g/bird	570	575	605	540	13.76	0.480	0.001	0.213
G:F, g/kg	621	648	688	581	10.80	0.008	0.001	0.382
Days 21 to 26								
BW gain, g/bird	386	418	423	381	8.29	0.004	0.001	0.059^3^
Feed intake, g/bird	604	626	653	578	15.90	0.250	0.001	0.861
G:F, g/kg	639	673	650	661	12.61	0.067	0.560	0.135
Days 14 to 26								
BW gain, g/bird	741	791	838	694	11.09	0.001	0.001	0.121
Feed intake, g/bird	1,174	1,202	1,258	1,118	25.39	0.235	0.001	0.566
G:F, g/kg	630	660	668	622	9.14	0.014	0.001	0.354

^1^Data are means of 16 replicate cages.

^2^Standard error of mean.

^3^The respective simple effect means for 0 g/kg Spirulina and no-challenge, 0 g/kg Spirulina and challenged, 5 g/kg Spirulina and no-challenge, and 5 g/kg Spirulina and challenged were 416, 356, 429, and 406.

BW = body weight; G:F = gain-to-feed ratio.

### Nutrient digestibility

Table 4 outlines the effect of an *Eimeria* challenge and Spirulina supplementation on the digestibility of nutrients in broiler chickens. The TTR of N increased (*P* < 0.05) with Spirulina supplementation on 6 dpi, regardless of the challenge state; the addition of Spirulina in the diet also increased (*P* < 0.01) the AME and AMEn of the diets fed to birds on 6 dpi. There was a tendency (*P* = 0.08) for dietary Spirulina to increase the TTR of GE and DM in birds. On 11 dpi, there was a Spirulina × Challenge interaction on the TTR of N (*P* < 0.05). Additionally, there was a Spirulina × Challenge interaction (*P* < 0.05) on the AME of diets fed to birds. Dietary Spirulina supplementation had no effect on the AID of nutrients. However, the *Eimeria* challenge significantly reduced (*P* < 0.05) the utilization of all nutrients and energy on 6 dpi and 11 dpi, irrespective of the diet.

**Table 4.** Nutrient digestibility of *Eimeria*-challenged broiler chickens fed Spirulina-supplemented diets, 6- and 11-dpi

Item	Spirulina, 0 g/kg		Spirulina, 5 g/kg		SD^1^	*P* value		
	No-challenge	Challenge	No-challenge	Challenge		Spirulina	Challenge	Spirulina × Challenge
6 dpi								
TTR of DM, %	71.6	51.5	72.3	54.2	10.12	0.078	0.001	0.306
TTR of GE, %	75.5	50.3	77.3	52.5	13.09	0.085	0.001	0.864
TTR of N, %	65.1	38.9	66.6	45.1	13.22	0.024	0.001	0.150
AME, kcal/kg DM	2,940	1,925	3,056	2,075	521.57	0.003	0.001	0.680
AMEn, kcal/kg DM	2,816	1,830	2,886	1,994	491.62	0.001	0.001	0.155
11 dpi								
TTR of DM, %	72.9	72.5	74.8	71.4	3.19	0.706	0.067	0.135
TTR of GE, %	77.4	76.3	79.0	75.3	2.70	0.701	0.006	0.119
TTR of N, %	72.4^a^	69.8^ab^	74.3^a^	66.1^b^	4.38	0.475	0.001	0.027
AME, kcal/kg DM	3,017^b^	2,971^b^	3,162^a^	2,978^b^	111.12	0.015	0.001	0.025
AMEn, kcal/kg DM	2,867	2,790	2,988	2,810	99.45	0.008	0.001	0.051
AID of DM, %	70.1	64.0	71.1	63.0	5.22	0.988	0.001	0.469
AID of GE, %	74.2	66.5	75.2	66.6	5.06	0.627	0.001	0.679
AID of N, %	79.5	73.5	81.4	73.2	4.87	0.500	0.001	0.382
AID of P, %	50.3	40.1	50.9	41.9	6.81	0.515	0.001	0.743
IDE, kcal/kg DM	2,890	2,592	2,974	2,635	200.90	0.147	0.001	0.637
No. of replicates	8	7	8	7				

^1^Standard deviation.

^a,b,c^Least squares means within a row without a common superscript differ at *P* < 0.05.

TTR = total tract retention; DM = dry matter; GE = gross energy; N = nitrogen; AME = apparent metabolizable energy; AMEn = nitrogen-corrected apparent metabolizable energy; AID = apparent ileal digestibility; IDE = ileal digestible energy.

### Intestinal histomorphology

There was no interaction of dietary Spirulina supplementation and *Eimeria* challenge on the intestinal histomorphology of birds (Table 5). On 6 dpi, the ileal villus perimeter and area both increased (*P* < 0.05) with dietary Spirulina supplementation. The villus height, crypt depth, and goblet cell count in the ileum were all increased (*P* < 0.05) by the *Eimeria* challenge. However, only the crypt depth was increased (*P* < 0.01) by the *Eimeria* challenge in the jejunum. The *Eimeria* challenge led to a decrease (*P* < 0.05) in VH/CD in both the ileum and jejunum on 6 dpi.

**Table 5.** Ileal and jejunal histomorphology in *Eimeria*-challenged birds fed Spirulina-supplemented diets, 6- and 11-dpi^1^

Item	Spirulina		*Eimeria* challenge		SEM^2^	*P* value		
	0 g/kg	5 g/kg	No-challenge	Challenge		Spirulina	Challenge	Spirulina × Challenge
Ileum, 6 dpi (µm)								
Villus height	570	535	508	597	25.44	0.251	0.008	0.944
Crypt depth	175	186	144	217	10.72	0.463	0.001	0.914
VH/CD	3.6	3.2	3.7	3.0	0.22	0.223	0.030	0.417
Villus perimeter	1,370	1,549	1,449	1,470	46.56	0.005	0.718	0.771
Villus area, mm^2^	0.10	0.12	0.11	0.12	0.01	0.038	0.181	0.948
Goblet cell count	144	127	115	156	11.96	0.264	0.010	0.213
Jejunum, 6 dpi (µm)								
Villus height	836	866	904	799	55.03	0.702	0.186	0.199
Crypt depth	254	262	172	344	14.38	0.708	0.001	0.605
VH/CD	4.0	4.3	5.8	2.4	0.33	0.502	0.001	0.473
Villus perimeter	1,917	1,973	2,002	1,888	114.43	0.730	0.488	0.421
Villus area, mm^2^	0.19	0.18	0.19	0.18	0.02	0.859	0.794	0.153
Goblet cell count	49	54	50	52	5.69	0.538	0.774	0.446
Ileum, 11 dpi (µm)								
Villus height	497	520	489	529	21.15	0.388	0.144	0.541
Crypt depth	143	137	116	164	7.78	0.580	0.001	0.571
VH/CD	3.7	4.1	4.3	3.5	0.20	0.154	0.005	0.882
Villus perimeter	1,288	1,474	1,398	1,364	66.81	0.029	0.676	0.950
Villus area, mm^2^	0.11	0.10	0.10	0.10	0.01	0.530	0.591	0.356
Goblet cell count	147	257	215	189	36.33	0.028	0.585	0.403
Jejunum, 11 dpi (µm)								
Villus height	820	853	922	751	39.68	0.571	0.005	0.365
Crypt depth	172	147	133	186	8.75	0.051	0.001	0.340
VH/CD	5.2	6.6	7.2	4.5	0.41	0.006	0.001	0.310
Villus perimeter	2,016	2,045	2,238	1,822	87.75	0.816	0.002	0.479
Villus area, mm^2^	0.19	0.20	0.20	0.19	0.02	0.488	0.751	0.702
Goblet cell count	40	44	38	47	5.18	0.527	0.134	0.742

^1^Data are means of 16 replicate cages.

^2^Standard error of mean.

VH/CD = villus height to crypt depth ratio.

On 11 dpi, dietary Spirulina addition increased (*P* < 0.05) the ileal villus perimeter and goblet cell count but not villus area. In the jejunum, the tendency (*P* = 0.051) for a reduction in crypt depth, resulted in an increased VH/CD due to dietary Spirulina supplementation (*P* < 0.01). The *Eimeria* challenge increased crypt depth in the ileum, leading to a decreased VH/CD (*P* < 0.01). A reduction in jejunal villus height and an increase in jejunal crypt depth resulted in a decreased VH/CD in challenged birds (*P* < 0.01). The jejunal villus perimeter was also reduced (*P* < 0.01) with the *Eimeria* challenge.

### Serum biomarkers, bone parameters, and oocyst count

Table 6 shows the serum biomarkers, bone parameters, and oocyst count of birds on 6 dpi and 11 dpi. Apart from the oocyst count, there was no interaction between the dietary Spirulina and the *Eimeria* challenge. On 6 dpi, dietary Spirulina addition increased (*P* < 0.05) catalase activity in the serum of birds. However, there was no dietary Spirulina effect on ALP, TAC, bone-breaking strength, bone ash, or oocyst count. The *Eimeria*-challenged birds had lower levels of serum ALP, catalase, TAC, bone-breaking strength, and elevated oocysts in the excreta (*P* < 0.05).

**Table 6.** Serum biomarkers, bone parameters, and oocyst count in *Eimeria*-challenged birds fed Spirulina-supplemented diets, 6- and 11-dpi^1^

Item	Spirulina		*Eimeria* challenge		SEM^2^	*P* value		
	0 g/kg	5 g/kg	No-challenge	Challenge		Spirulina	Challenge	Spirulina × Challenge
6 dpi								
ALP, U/L	140.0	139.8	140.5	139.2	0.22	0.457	0.001	0.290
Catalase, U/L	27.2	33.3	33.6	27.0	1.69	0.016	0.010	0.621
TAC, mM	1.6	1.7	2.1	1.1	0.16	0.595	0.001	0.446
Bone-breaking strength, N	149.9	156.1	165.9	140.0	9.85	0.524	0.013	0.802
Bone ash, %	51.3	51.1	51.6	50.8	0.56	0.836	0.373	0.910
Oocyst count, log_10_	2.7	2.7	-	5.4	0.01	0.496	0.001	0.501
11 dpi								
ALP, U/L	766.7	860.5	784.1	843.1	81.34	0.422	0.612	0.751
Catalase, U/L	11.1	15.0	14.9	11.2	1.04	0.013	0.019	0.108
TAC, mM	1.7	2.1	2.3	1.5	0.12	0.014	0.001	0.846
Bone-breaking strength, N	188.4	193.2	213.2	168.3	10.86	0.599	0.001	0.523
Bone ash, %	50.2	55.3	57.9	47.6	1.67	0.025	0.001	0.228
Oocyst count^3^, log_10_	2.6	2.5	-	5.1	0.02	0.024	0.001	0.015

^1^Data are means of 16 replicate cages.

^2^Standard error of mean.

^3^Interaction means are as follows: 0.0, 5.2, 0.0, and 5.0 for non-challenged birds fed control diet, challenged birds fed control diet, non-challenged birds fed Spirulina-supplemented diet, and challenged birds fed Spirulina-supplemented diet, respectively.

ALP = alkaline phosphatase; TAC = total antioxidant capacity; N = Newton.

On 11 dpi, dietary Spirulina not only increased (*P* < 0.05) the production of serum catalase but also TAC. Additionally, there was increased (*P* < 0.05) ash deposition in the tibia of birds with dietary Spirulina supplementation. An observed reduction in oocyst count in the excreta of Spirulina-fed challenged birds on 11 dpi resulted in a significant Spirulina × Challenge state interaction (*P* < 0.05). Compared with the non-challenged birds, challenged birds had lower (*P* < 0.05) serum TAC, catalase, bone strength, and bone ash on 11 dpi.

### Gene expression

Table 7 shows the effect of Spirulina supplementation and an *Eimeria* challenge on jejunal mRNA expression in broiler chickens; there was no interaction between dietary Spirulina supplementation and *Eimeria* challenge. On 6 dpi, the relative mRNA expression of HMOX1 and SOD1 in jejunal mucosa was increased (*P* < 0.05) with dietary Spirulina supplementation. The challenged birds had higher (*P* < 0.05) relative mRNA expression of IL-6, IL-1β, TNFα, and claudin 1 in their jejunal mucosa.

**Table 7.** Jejunal mRNA expression in *Eimeria*-challenged birds fed Spirulina-supplemented diets, 6- and 11-dpi

Item	Spirulina		*Eimeria* challenge		SD^1^	*P* value		
	0 g/kg	5 g/kg	No-challenge	Challenge		Spirulina	Challenge	Spirulina × Challenge
6 dpi								
HMOX1	1.07	1.50	1.21	1.36	0.44	0.009	0.351	0.962
IL-1β	1.39	1.53	1.00	1.91	0.89	0.605	0.002	0.566
IL-6	1.15	1.26	0.99	1.41	0.61	0.540	0.027	0.442
SOD1	0.94	1.20	1.07	1.07	0.33	0.033	0.949	0.171
TNFα	1.27	1.43	1.08	1.63	0.49	0.270	0.001	0.963
Claudin 1	2.20	2.18	0.98	3.39	1.59	0.946	0.001	0.966
11 dpi								
HMOX1	0.84	1.04	1.09	0.79	0.32	0.041	0.005	0.801
IL-1β	0.92	1.03	1.06	0.89	0.40	0.438	0.228	0.913
IL-6	0.92	0.89	0.89	0.92	0.43	0.885	0.815	0.184
SOD1	0.97	1.10	1.04	1.03	0.20	0.037	0.850	0.321
TNFα	1.01	1.26	1.13	1.15	0.22	0.002	0.778	0.928
Claudin 1	0.90	1.63	1.28	1.25	0.82	0.014	0.935	0.567
No. of replicates	14	15	15	14				

^1^Standard deviation.

HMOX1 = heme oxygenase 1; IL-1β = interleukin 1 beta; IL-6 = interleukin 6; SOD1 = superoxide dismutase 1; TNFα = tumor necrosis factor alpha.

In a similar pattern, dietary Spirulina addition increased (*P* < 0.05) the relative mRNA expression of HMOX1 and SOD1 in the jejunal mucosa of birds on 11 dpi; the relative mRNA expression of claudin 1 and TNFα was also increased (*P* < 0.01). The *Eimeria*-challenged birds had a lower mRNA expression (*P* < 0.05) of jejunal HMOX1 relative to non-challenged birds.

## Discussion

### Growth performance

This study aimed to investigate the effect of dietary Spirulina supplementation on the growth performance, nutrient utilization, intestinal integrity, immune response, and serum biomarkers in *Eimeria*-challenged birds. The observed reduction in BW gain and FI on 6 and 11 dpi confirms the results of various studies that have reported the negative effect of an *Eimeria* challenge on the growth performance of birds (Osho and Adeola, 2019; Lin and Olukosi, 2021; Li et al., 2023). Dietary Spirulina has been shown to improve the BW gain, FI, and feed conversion rate of birds (Park et al., 2018; Moustafa et al., 2021). These previous studies mainly focused on supplementing the diet of healthy or heat-stressed birds with Spirulina. In contrast, the current study used an *Eimeria* challenge model to evaluate the effects of dietary Spirulina on birds. The supplementation of Spirulina had positive effects on the BW gain and G:F of birds, regardless of their challenge state. On 11 dpi, birds in the challenge group fed a Spirulina-supplemented diet tended to gain as much weight as birds in the no-challenge group that received the same diet. This tendency to regain BW due to dietary Spirulina addition could be attributed to the immune-modulating properties of this feed additive (Wu et al., 2016), which may help in mitigating the adverse impacts of parasitic infection on muscle accretion in birds.

Regardless of the challenge state, we observed improvement in the BW gain and G:F from days 14 to 26, highlighting the prolonged benefits of Spirulina addition to the diet of broiler chickens. The active components of Spirulina appear to positively impact metabolic systems linked to growth performance, such as the antioxidant and immune system (Park et al., 2018), leading to increased BW gain and G:F.

### Nutrient digestibility

Liu et al. (2019) and Abdel-Wahab et al. (2023) have indicated that Spirulina can stimulate proteolytic digestive enzymes in quails and promote *Lactobacillus* proliferation in pigs, potentially contributing to increased N digestion. The increased utilization of DM, GE, and N due to dietary Spirulina supplementation may have contributed to the increased growth performance in birds observed in this study. The increased AME and AMEn in diets supplemented with Spirulina on 6 dpi suggest increased energy utilization needed to support the growth of birds. Algae-derived polysaccharides and algae prebiotics have been demonstrated to impact energy utilization by influencing intestinal microbiota composition (De Jesus Raposo et al., 2016; Cheng and Kim, 2022).

On 11 dpi, non-challenged birds that received a Spirulina-supplemented diet had higher TTR of N than challenged birds that received a Spirulina-supplemented diet. Notably, the TTR of N decreased by approximately 11% in challenged birds on Spirulina-supplemented diets. This result shows that while Spirulina supplementation may improve N digestion in non-challenged birds, it may not mitigate the negative impact of an *Eimeria* challenge on N utilization in birds on the same diet. Notwithstanding, the decline in the TTR of N in these birds warrants further scientific inquiry. Still on 11 dpi, energy utilization did not differ significantly across the experimental groups, except for non-challenged birds fed dietary Spirulina, where the AME was more than 5% higher. These results suggest that the positive impact of Spirulina on nutrient utilization is more pronounced in healthy birds than in those challenged by *Eimeria* on 11 dpi. There was no significant effect of Spirulina on the AID of nutrients; this is supported by the lack of dietary Spirulina influence on ileal villus height, crypt depth, and villus area on 11 dpi. The *Eimeria* challenge reduced the digestibility of all nutrients on 6 and 11 dpi, underscoring the detrimental effect of *Eimeria* parasites on the proper utilization of nutrients in birds (Adedokun et al., 2016; Yuan et al., 2022).

### Intestinal histomorphology

The contribution of dietary Spirulina supplementation in improving growth performance may be attributed to increased gut surface area, leading to efficient nutrient absorption on 6 dpi. As Ravindran et al. (2006) described, an increase in surface area likely corresponds to increased nutrient digestion. The greater villus area in birds may further elevate digestive enzyme activity, leading to increased growth on 6 dpi (Ravindran and Abdollahi, 2021). Moreover, on 11 dpi, birds that were fed Spirulina-supplemented diets had higher ileal villus perimeter and goblet cells compared with birds without dietary Spirulina. However, this did not translate to improvement in the TTR of DM, GE, and N. It is worth noting that the increase in ileal goblet cell counts on 11 dpi indicates increased mucin production, which, in turn, can contribute to the integrity of the intestinal mucus barrier in birds (Specian and Oliver, 1991; Almeida et al., 2014). On 11 dpi, the higher jejunal VH/CD in birds fed dietary Spirulina aligns with the notion of reduced turnover of the intestinal mucosa (Laudadio et al., 2012), which may result in lower energy maintenance requirements. This may have also contributed to the increased growth performance of birds observed from days 21 to 26. Taken together, these results show that the favorable effects of incorporating Spirulina into the dietary regimen of birds extend beyond growth performance, regardless of the challenge state of birds. They underscore the significance of dietary Spirulina supplementation in optimizing the intestinal milieu of birds irrespective of their challenge state.

On another note, the deeper crypts in the ileum and jejunum of *Eimeria*-challenged birds on 6 dpi suggest an elevated intestinal epithelium turnover accompanied by an increased metabolic cost. This observation is supported by the *Eimeria*-induced reduction in VH/CD observed in the ileum and jejunum of birds (Gautier et al., 2020). Consequently, the reduction in BW gain and G:F in challenged birds on 6 dpi may be linked to these gastrointestinal changes. However, the greater ileal villus height on 6 dpi in the *Eimeria*-challenged birds is abnormal. It has been established that *Eimeria* challenge leads to villus atrophy and the destruction of the epithelial linings of the intestine and not the other way around (Pout, 1967; Calik et al., 2019). This observation underlines a key point: when evaluating gut health in birds undergoing an *Eimeria* challenge, it is essential not to consider villus height in isolation. Instead, all histomorphology metrics should be interpreted collectively for a comprehensive assessment. Additionally, an increase in the number of ileal goblet cells in challenged birds on 6 dpi may be attributed to the presence of pathogenic substances which prompts an intestinal mucogenic reaction. Moreover, *Eimeria* parasites may induce local T cell-mediated inflammatory responses in *Eimeria*-challenged broiler chickens, leading to elevated mucin production (Collier et al., 2008).

### Serum biomarkers, bone parameters, and oocyst count

We observed positive effects of Spirulina supplementation on the antioxidant status of birds on 6 dpi and 11 dpi, regardless of *Eimeria*-challenge state. Catalase activity was increased in birds fed Spirulina-supplemented diets, indicating an increase in the removal of free radicals that can cause cellular damage (Nishikawa et al., 2009). This is supported by the increased TAC in birds. Spirulina has been shown to increase the levels of antioxidant biomarkers in broilers. This positive effect on the antioxidant system is attributed to specific components in Spirulina, such as phycocyanin, polyunsaturated fatty acids, and polysaccharides (Han et al., 2021). These components play a role in enhancing the antioxidant mechanisms in birds.

Currently, there is a lack of information regarding the effects of dietary Spirulina on bone ash in broiler chickens. Also, it is important to note that the rapid growth in broiler chickens may significantly affect their musculoskeletal integrity (Julian, 1998; Rath et al., 2000). Nevertheless, our study demonstrated an increase in tibia bone ash in birds that received Spirulina-supplemented diets on 11 dpi, although this effect was not present on 6 dpi. This observation indicates an improvement in bone mineralization, which contributes to the overall skeletal strength of the birds. Additionally, Spirulina has moderate vitamin B_12_ content, which has been associated with stimulating osteoblastic proliferation and increasing 25-OH vitamin D levels, both of which promote bone health and remineralization (Ekeuku et al., 2022). In the same vein, this study is among the first to investigate the impact of dietary Spirulina supplementation on oocyst replication in birds. Dietary Spirulina addition led to reduced oocyst shedding in challenged birds, potentially contributing to the reduced intestinal damage observed. While the specific mode of action remains unclear, Spirulina has been shown to cause deformities in proglottids and swelling of the scolex in parasitic worms, ultimately impairing tegument function (Al-Otaibi et al., 2021). These findings suggest the potential antiparasitic effects of Spirulina.

Furthermore, it is known that birds naturally produce reactive oxygen species as part of their defense mechanism. However, coccidiosis can lead to an increase in reactive oxygen species production (Ponnampalam et al., 2022). This elevation in reactive oxygen species levels may disrupt the antioxidant system, possibly compromising the effectiveness of serum antioxidant enzymes like catalase and the TAC, as observed in the challenged birds. On another note, the decreased serum ALP, bone ash, and bone-breaking strength among the *Eimeria*-challenged birds indicate a potential issue with absorbing crucial minerals and vitamins essential for bone health (Tompkins et al., 2023). This reduction in bone-related response criteria may be attributed to the oxidative stress induced by the *Eimeria* challenge, leading to a disruption in bone homeostasis. Consequently, this disruption can negatively impact bone quality and mineral content (Tompkins et al., 2022, 2023).

### Gene expression

The elevated mRNA expression of antioxidant genes (HMOX1 and SOD1) observed in the jejunum of birds fed dietary Spirulina on 6 dpi and 11 dpi aligns with the increased serum antioxidant enzyme levels. HMOX1 is an important antioxidant gene, and cells lacking its expression are more susceptible to oxidative damage. The increase in the relative mRNA expression of HMOX1 may aid in mitigating the rise in free radicals during infections or stress (Poss and Tonegawa, 1997). Additionally, the SOD1 gene is responsible for the SOD enzyme production which guards against reactive oxygen species damage. It plays a protective role in the intestinal growth and health of birds (Fridovich, 1995; Tang et al., 2019; Abdel-Latif et al., 2021). The antioxidant properties of Spirulina have been previously demonstrated in broiler chickens. The various antioxidant compounds present in Spirulina, such as β-carotene, phycocyanin, phenolic compounds, and tocopherol, have the potential to improve the overall antioxidant status of birds (Park et al., 2018; Abdel-Moneim et al., 2020; Moustafa et al., 2021). Furthermore, studies have indicated that Spirulina may increase the mRNA expression of antioxidant genes in other animals like hyperglycemic rats and rainbow trout (Sadek et al., 2017; Teimouri et al., 2019). Although ongoing research explores additional pathways influenced by Spirulina, it is known that the 2 most active antioxidants in Spirulina, phycocyanin, and β-carotene, may regulate pathways like ERK1/2, JNK, and p38, potentially leading to the activation of downstream antioxidant and inflammatory genes (Wu et al., 2016).

Anderson and Van Itallie (2009) have noted that preserving the integrity of tight junctions is crucial for safeguarding the gut against antigens and infections that could otherwise cause intestinal inflammation and damage. Studies have shown that a purified Spirulina polysaccharide can enhance the tight junctions in the intestine and maintain the integrity of epithelial tight junctions (Wang et al., 2020). Our study also supports this observation as shown by the increased mRNA expression of claudin 1 in the jejunal mucosa of birds fed dietary Spirulina, regardless of their *Eimeria*-challenge state. The increase in the relative mRNA expression of TNFα in birds fed Spirulina-supplemented diets is associated with the immunomodulatory properties of Spirulina, indicating an activation of the immune system in birds (Shokri et al., 2014). As anticipated, the *Eimeria* challenge increased the relative mRNA expression of inflammatory gene markers in the jejunum of birds on 6 dpi. Also, the abnormal alterations observed in claudin 1 mRNA expression in challenged birds are consistent with reported effects of *Eimeria* infection, which induces acute inflammation and disrupts tight junctions in birds (Xu et al., 2016; Teng et al., 2020).

## Conclusion

In conclusion, dietary Spirulina increased the TTR of N on 6 dpi, possibly by stimulating digestive enzymes in birds. Additionally, dietary Spirulina positively influenced antioxidant mRNA expression in birds, enhanced their immune response, and maintained intestinal integrity. These findings shed light on the multifaceted effects of dietary Spirulina on broiler chicken health, suggesting its potential as a valuable dietary supplement.

## Abbreviations

AID: apparent ileal digestibility
ALP: alkaline phosphatase
AME: apparent metabolizable energy
AMEn: nitrogen-corrected apparent metabolizable energy
AOAC: Association of Official Analytical Chemists
BW: body weight
cDNA: complementary deoxyribonucleic acid
DM: dry matter
DPI: days post infection
ERK1/2: extracellular signal-regulated kinase1/2
FI: feed intake
G:F: gain-to-feed ratio
GAPDH: glyceraldehyde-3-phosphate dehydrogenase
GE: gross energy
HMOX1: heme oxygenase 1
IL-10: interleukin 10
IL-1β: interleukin-1beta
IL-6: interleukin 6
JNK: c-Jun amino-terminal kinase
mRNA: messenger ribonucleic acid
N: nitrogen
PCR: polymerase chain reaction
RNA: ribonucleic acid
ROS: reactive oxygen species
SOD: superoxide dismutase
SOD1: superoxide dismutase type 1
TAC: total antioxidant capacity
TNFα: tumor necrosis factor alpha
TTR: total tract retention
VH/CD: villus height to crypt depth ratio
